# Enhancing Gluten-Free Crispy Waffles with Soybean Residue (Okara) Flour: Rheological, Nutritional, and Sensory Impacts

**DOI:** 10.3390/foods13182951

**Published:** 2024-09-18

**Authors:** Aunchalee Aussanasuwannakul, Kassamaporn Puntaburt, Thidarat Pantoa

**Affiliations:** 1 Department of Food Chemistry and Physics, Institute of Food Research and Product Development, Kasetsart University, Bangkok 10903, Thailand; 2 Department of Food Processing and Preservation, Institute of Food Research and Product Development, Kasetsart University, Bangkok 10903, Thailand

**Keywords:** food waste valorization, circular economy, food sustainability, texture analysis, viscoelasticity, Check-All-That-Apply, cholesterol adsorption, glucose adsorption, dietary fiber, protein enrichment

## Abstract

The incorporation of okara, a by-product of soybean milk production, into gluten-free products such as crispy waffles poses challenges due to the absence of gluten’s viscoelastic properties and the high fiber content of okara. This study aimed to evaluate the effects of okara flour on the rheological properties, physical attributes, and sensory qualities of gluten-free waffles. Waffle batters with varying levels of okara flour (10%, 20%, 30%, and 40%) were prepared, and their rheological properties were analyzed using oscillatory shear and creep-recovery tests. Physical properties, proximate composition, cholesterol and glucose adsorption capacities, storage stability, and sensory attributes were also assessed. The results demonstrated that increasing okara flour content improved batter elasticity and viscosity (with complex viscosity reaching up to 10,923 Pa·s for 40% okara flour) but decreased spread ratio by up to 45% and increased moisture content by approximately 2.7%. Higher okara content also led to a 16% decrease in brightness (L*) and increased hardness, reaching 325.26 g/s at 40% substitution. Sensory evaluation revealed that waffles with 30% okara flour were preferred for their texture and overall liking, with a score of 7.43 compared to higher substitution levels. Cholesterol and glucose adsorption capacities were high in okara flour, contributing to potential health benefits. Storage stability tests showed acceptable moisture content, water activity, and microbiological safety over 60 days, though hardness decreased by about 42%. In conclusion, okara flour enhances the nutritional profile of gluten-free waffles, but its impact on texture and flavor requires careful formulation adjustments to optimize consumer acceptance.

## 1. Introduction

The tofu industry produces approximately 1.4 billion tons of tofu dreg (okara) annually, predominantly in Asian countries [1]. In Thailand, the estimated production of fresh okara is around 8.04 million tons annually [2]. High in nutrients and moisture, okara quickly undergoes putrefaction, necessitating proper management to prevent environmental problems [3].

Our research focuses on recycling okara using simple technology, such as tray drying and grinding, which is practical and widely beneficial for the soymilk and tofu processing industry, particularly for medium and small-scale enterprises. We demonstrate the feasibility of converting okara into high-value flour (moisture content < 10%, water activity < 0.3, and high water-holding and swelling capacities) at a pilot plant level (tray drying at 100 °C), handling at least 3% of the total okara produced (a projected 911 tons per year from 306 small-scale processors in Bangkok) [4]. Ginting et al. (2024) [5] reported the significant added value of okara products, from 0.40 times for flour to 582 times for crackers, highlighting their potential contribution to small-scale food processor income and food waste recycling.

Flour making and subsequent flour processing into food products is crucial for small-scale processors, as the added value of okara-based products can significantly increase income, transforming a low-yield flour production process into a profitable venture through the creation of diverse, high-quality, nutritious, and marketable food items. The potential use of okara flour as a food ingredient and value-added product high in protein and fiber is well supported in plant-based and gluten-free concepts. For instance, blending okara with mung beans and rice in various proportions has resulted in nutritionally rich and sensorially pleasing snacks, as shown in the study by Aussanasuwannakul et al. (2023) [6]. Additionally, a study on the valorization of soybean residue (okara) demonstrated that okara flour, both as oil and defatted powder, possesses enhanced nutritional and functional properties, making it a high-potential, high-value food ingredient [7].

However, okara flour is less suitable for products requiring foaming action, such as brownies, cakes, and bread, due to its inability to form a gluten network, which is essential for maintaining texture and volume. The high dietary fiber content in okara flour absorbs water quickly and strongly, disrupting gas-holding capacity and starch gelatinization during baking, leading to smaller loaf volumes and denser textures. Pešić et al. (2023) [8] found that okara-enriched gluten-free bread had poorer textural properties compared to traditional gluten-containing bread, while Ginting et al. (2024) [5] reported similar issues in brownies and cakes, where the lack of gluten network resulted in compromised product quality. These studies indicate that okara flour, while nutritionally beneficial, presents significant technological challenges in baked goods that require foaming action.

To overcome such limitations, okara flour from basic processing technology (tray drying) has been successfully tested in extruded snacks, where its high protein and fiber content, combined with complementary functions of protein and starch from other ingredients, contribute to improved physicochemical properties, texture, and mouthfeel. A study by Aussanasuwannakul et al. (2022) [6] demonstrated that blending okara with mung bean and rice flours, along with corn grit, creates a high-protein (>15%), high-fiber (22%), gluten-free snack with optimized properties, highlighting the significant value-added potential of okara in plant-based and gluten-free products.

Given the complementary function of proteins and starch, another promising application for okara flour is in the production of crispy waffles. Unlike brownies, cakes, and bread, crispy waffles do not rely on a foaming action for their texture. Instead, they achieve their firm, crunchy texture through high heat and low moisture, reducing the need for hydrocolloids to manage water content. Similar to crispy waffles, ice cream cones made from composite flour blends of wheat and okara have shown improved product characteristics. Fatmawati et al. (2023) [9] found that ice cream cones with 20% okara flour had the highest acceptance in terms of color, taste, aroma, and crispness. Incorporating fiber-rich agricultural materials like okara can increase the protein and fiber content while benefiting from the structural changes induced by high-temperature cooking. This process improves the sensory qualities of the final product, such as flavor, color, and texture, and extends shelf life, aligning with consumer demands for healthier, sustainable crispy snacks.

In terms of polysaccharides, okara is a good source of dietary fiber, ranging from 13.43 to 76.38% (fresh weight) and 56.6 to 58.6% (dry weight), with the main proportion being insoluble dietary fiber, such as cellulose [5]. Dietary fiber is a functional compound that plays an essential role in the physiological processes of the human body and is associated with preventing some diseases. High-temperature cooking, such as that used in making crispy waffles, can enhance the technological properties of okara by affecting its dietary fiber composition, leading to increased sugar and fat adsorption. This modification not only improves the textural qualities of food products but also promotes health benefits by potentially lowering blood cholesterol and glucose levels, thereby reducing the risk of coronary heart disease, diabetes, and obesity.

The primary objective of this study is to investigate the feasibility and effectiveness of utilizing okara flour, a by-product of soybean milk processing, in the development of crispy waffles. Specifically, the study aims to evaluate the rheological properties of waffle batter with varying levels of okara flour substitution, assess the physical and textural characteristics, and determine the nutritional benefits of incorporating okara flour, focusing on protein and fiber content. Additionally, the study will analyze the sensory attributes and consumer acceptance of crispy waffles containing okara flour.

This research holds significant implications for both the food industry and environmental sustainability. By recycling okara into high-value food products, it contributes to reducing food waste and promoting sustainable food production practices. Okara flour, being rich in protein and fiber, can enhance the nutritional profile of crispy waffles, aligning with consumer demands for healthier snack options. Furthermore, the added value of okara-based products can increase income for small and medium-scale food processors, transforming a low-yield by-product into a profitable venture.

We hypothesize that increasing the substitution level of okara flour in waffle batter will improve the nutritional value of the waffles but may affect their textural properties. Additionally, it is expected that waffles with higher okara flour content will have higher protein and fiber content compared to those with lower or no okara flour. To test these hypotheses, the study will explore how okara flour substitution affects the rheological properties of waffle batter, the physical and textural differences in the final product, and the sensory attributes and levels of consumer acceptance for crispy waffles containing different amounts of okara flour.

## 2. Materials and Methods

### 2.1. Materials

The full-fat okara flour used in this study was obtained as a byproduct of the pilot soymilk processing line at the Institute of Food Research and Product Development, Kasetsart University, Bangkok, Thailand; 3.8% moisture, 36% protein, 2.9% fat, and 55% carbohydrate. Fresh okara was tray dried, pulverized, and sieved through a 60-mesh sieve, following the method described in our previous research [6]. Glutinous rice flour (Twin Gold brand, Thai Flour Industry Co., Ltd., Bangkok, Thailand; 8.8% moisture, 6.6% protein, 0.4% fat, 83% carbohydrate) and tapioca flour (Ton Son brand, Sitthinan Co., Ltd., Pathum Thani, Thailand; 12% moisture, 0.3% protein, 0.1% fat, 87% carbohydrate), coconut milk (Chaokoh brand, Theppadungporn coconut Co., Ltd., Nakhon Pathom Thailand), cocoa powder (Tulip brand, Freyabadi Indotama Company, Jakarta Indonesia), bakery sugar (Mitrphol brand, Mitr Phol Sugar Corporation., Ltd., Bangkok Thailand), salt (Prangthip brand, Saha Pathanapibul PLC., Bangkok Thailand), and baking powder (Imperial brand, KCG Corporation Public Co., Ltd., Bangkok Thailand) were also used.

### 2.2. Preparation of Waffles

**Figure 1:**
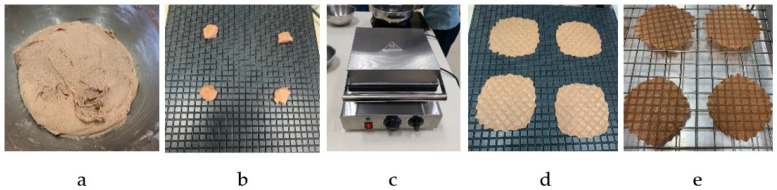
Preparation process and equipment for okara-added crispy waffles: (**a**) batter, (**b**) weighed batter on hot plate, (**c**) waffle maker, (**d**) cooked waffle, and (**e**) cooled waffle.

Okara flour was used as a substitute for glutinous rice flour in varying proportions: 10%, 20%, 30%, and 40% by weight. The waffle batter was prepared using the recipe detailed in Table 1. The process began with thoroughly mixing the dry ingredients, followed by gradually adding coconut milk until a homogenous batter was achieved (Figure 1a). After allowing the batter to rest for 5 min, 4 g portions were placed onto a preheated waffle maker set at approximately 125 °C (Figure 1b). The batter was then cooked in the waffle maker (Figure 1c). Each side of the waffle was baked for one minute to ensure even cooking and a crisp texture (Figure 1d). Post-baking, the waffles were carefully removed and allowed to cool to room temperature (Figure 1e). For storage, the waffles were placed in laminated aluminum foil pouches to maintain their quality and crispness until further analysis. The 12 × 20 cm^2^ laminated foil pouches were made from a laminated film consisting of polyethylene terephthalate, aluminum, and polyethylene with a zip lock.

### 2.3. Rheological Analysis of Waffle Batter

#### 2.3.1. Sample Preparation

**Figure 2:**
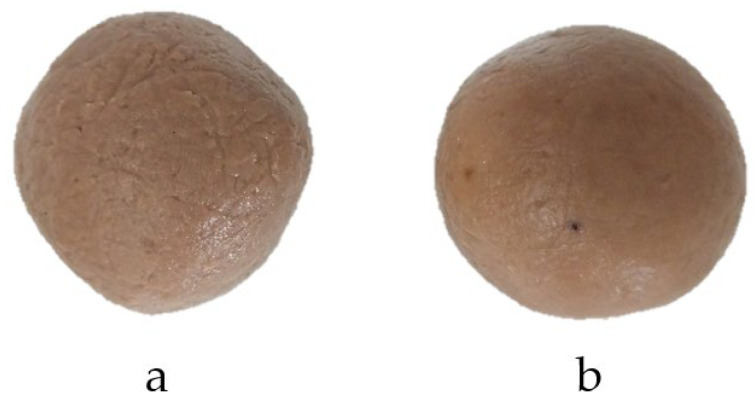
Waffle batter prepared by replacing glutinous rice flour with 40% okara flour at two particle size ranges: (**a**) 60 mesh and (**b**) 80 mesh, for rheological analysis.

During the waffle batter preparation process, a homogeneous portion was reserved for rheological analysis. This analysis focused on four samples prepared from two treatments: okara flour replacement levels (30% and 40%) and particle size ranges (60 mesh and 80 mesh) for the 40% replacement level. The different particle sizes were selected to assess the influence of flour fineness on the batter’s rheological properties, as finer particles are expected to enhance the structural integrity and consistency of the batter. The batter was kneaded until it achieved a smooth and homogeneous consistency and was then refrigerated at 4 °C overnight prior to testing. Figure 2 illustrates the batter’s appearance post-preparation.

#### 2.3.2. Oscillatory Measurements

**Figure 3:**
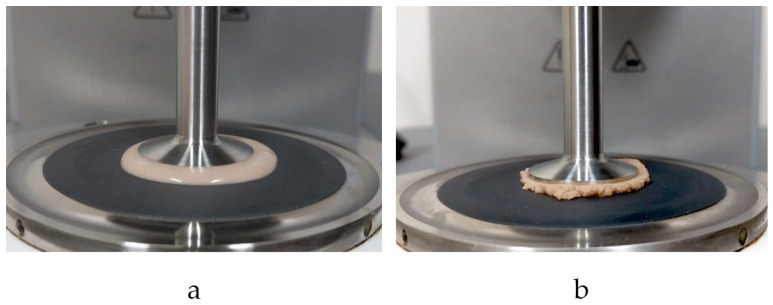
Filling of the plate/plate measuring system of the MCR302 during gap setting with waffle batter: (**a**) control and (**b**) 30% okara flour replacement.

The dynamic oscillatory shear measurements of the viscoelastic properties of the samples followed those previously reported by Aussanasuwannakul et al. [10] using a modular compact rheometer (Model MCR 302; Anton Paar, Graz, Austria) with parallel-plate geometry, a 25 mm diameter for the upper plate, and a gap width of 1 mm. The linear viscoelastic (LVE) range was determined using amplitude sweep tests before starting frequency sweep tests. To understand the structural character of the samples, amplitude sweeps were carried out by implementing a logarithmic increase of the strain (γ) from 0.01% to 100% with six measuring points per decade and a constant angular frequency (ω) of 10.0 rad/s. The temperature was set to remain constant at 25 °C. Storage moduli (G′) and loss moduli (G″) were recorded and analyzed using RheoCompass^TM^ software (Version 1.30, Anton Paar GmbH, Graz, Austria). The LVE range within which the G′ and G″ ran parallel to the *x*-axis was identified. The structural strength of the sample batter was expressed as the G′ value within the LVE range. Further, the limiting value of the LVE range ($\gamma_{L}$) was the strain value at which the G′-curve begins to deviate from the LVE plateau with the range of the tolerated deviation at 5%. The flow point (%) at crossover point where G′ = G″ was also identified. A frequency sweeps was performed at 25 °C with ω = 100.0–0.1 rad/s with three measuring points per decade in a pre-determined linear viscoelastic range at γ = 0.01% or 0.05% predicted for each sample to investigate the time-dependent deformation behaviors of the samples. Loss factor or damping factor tanδ, which is calculated as the G″/G′, reveals the ratio of the viscous and the elastic portion of the viscoelastic deformation behavior. Complex viscosity (η*) signified the viscoelastic flow resistance of the sample. The values of tanδ, and η* were recorded at 10 rad/s. Figure 3 shows the filling of the plate/plate measuring system of the MCR302 during gap setting with waffle batter.

#### 2.3.3. Transient Measurements

Creep and recovery tests were conducted to analyze the viscoelastic behavior performing two shear-stress steps following the procedure explained by [11] with slight modification. Using the same geometry and test condition as the oscillatory tests, the creep and recovery test preset for okara waffle dough included the following phases. (1) Stress phase: step in stress to $\tau_{0}$ = 10 Pa; this stress value was kept constantly for t = 5 min. (2) Rest phase: step to τ = 0 Pa; the sample remained unstressed for t = 5 min. As a test result, the time-dependent deformation function γ(t) was measured. The shear compliance J(t) can be calculated as a ratio between γ(t) and $\tau_{0}$. The final values of the reformation $\gamma_{e}$ and the permanently remaining deformation $\gamma_{v}$ were calculated from the curve by RheoCompass^TM^ software (Version 1.30, Anton Paar GmbH, Graz, Austria). The $\gamma_{max}$ is the maximum deformation, at the end of the stress phase; the $\gamma_{e}\;$ is the extent of reformation after the creep recovery phase, representing the elastic portion of the viscoelastic behavior; and the $\gamma_{v}$ is the extent of remaining deformation after the creep recovery phase, representing viscous portion [11]. The value of zero-shear viscosity $\eta_{0}$ at the end of the stress phase, when reaching steady-state flow behavior, was also calculated by the software.

### 2.4. Physical and Textural Properties of Crispy Waffles

#### 2.4.1. Spread Ratio Analysis

This method was adapted from the protocol established by the American Association of Cereal Chemists International (2010) [12] for measuring the spread ratio of cookie products. The analysis involves measuring the width and thickness of the waffle product in centimeters, with six pieces tested per sample. The spread ratio (SR) is calculated using the formula:

$$\mathrm{Spread}\;\mathrm{Ratio}\;(\mathrm{SR})=\frac{Width\;(cm)}{Thickness\;(cm)}$$

The width is measured by aligning six pieces side by side without overlapping and dividing the total width by six to get the average width per piece. Similarly, the thickness is determined by stacking six pieces and measuring the total height, then dividing by six for the average thickness per piece. Each formulation’s measurements are conducted in five replications. Results are expressed as mean and standard deviation.

#### 2.4.2. Water Activity (a_w_) and Moisture Content

The a_w_ of the samples was analyzed using a water activity meter (Model LabMaster-aw; Novasina, Zurich, Switzerland). The moisture content of the samples was analyzed following AOAC standard procedures [6], using the gravitational difference method involving samples dried at 105 °C, weighed pre- and post-cooling, with moisture content calculated and reported as mean and standard deviation across triplicates.

#### 2.4.3. Surface Color

The sample’s color was assessed using a HunterLab XE-Spector colorimeter (Hunter Associates Laboratory, Reston, VA, USA). Color parameters were represented as L*, a*, and b* values. The L* value indicated brightness ranging from white to black, the a* value represented the redness to greenness continuum (positive values for redness and negative values for greenness), and the b* value indicated the yellowness to blueness continuum (positive values for yellowness and negative values for blueness) on the Hunter meter. Prior to testing the samples, the colorimeter was calibrated using a standard white tile (porcelain).

#### 2.4.4. Proximate Composition and Nutritional Value

The protein, fat, ash, and total carbohydrate contents of the sample were determined following the methods described in [6]. The contents of total dietary fiber (TDF), insoluble dietary fiber (IDF), and soluble dietary fiber (SDF) in the sample were assayed using the enzymatic-gravimetric method of AOAC 985.29, AOAC 991.42, and AOAC 993.19, respectively, as described in [13].

#### 2.4.5. Texture Analysis

The hardness of okara waffles was analyzed using a Texture Analyzer (Model TA-XTplus^®^, Stable Micro Systems Ltd., Godalming, Surrey, UK) equipped with a P/0.25S ball probe (Stable Micro Systems Ltd.’s application study, REF: DOR1/CFS). The samples were tested 12 times, and the results were reported as mean values and standard deviations. The following test parameters were used: pre-test speed of 1.0 mm/s, test speed of 1.0 mm/s, post-test speed of 10.0 mm/s, distance of 3 mm, and trigger type set to Auto with a 5 g trigger force.

Sample preparation involved selecting the most uniform samples in terms of size and shape, which were removed from their packaging just prior to testing. Each sample was positioned centrally under the ball probe, which was lowered to a distance of 3 mm above the sample surface before the test commenced. The ball probe applied a force to the sample until the pre-determined distance was reached, and the force required to achieve this deformation was recorded.

Data were recorded and analyzed using Texture Exponent software (version 3.0.5.0; Stable Micro Systems Ltd., Godalming, Surrey, UK). The maximum force required to deform the sample was recorded as the hardness. The results from the 12 replicates were averaged, and the standard deviation was calculated to provide a measure of variability in the hardness of the okara waffles.

**Figure 4:**
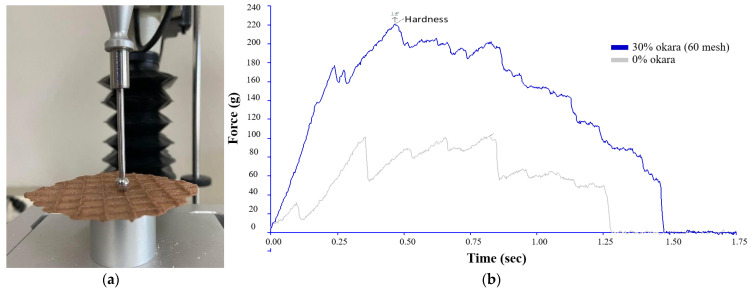
(**a**) Instrumental setup for the hardness test using a ball probe mounted on a TA.XTplus^®^ Texture Analyzer. (**b**) Schematic representation of a typical force–deformation curve for hardness, showing the peak force that indicates the hardness of the okara waffle samples.

As illustrated in Figure 4, the instrumental setup for the hardness test included a ball probe mounted on a TA.XTplus^®^ Texture Analyzer (Stable Micro Systems Ltd., Godalming, Surrey, UK) (Figure 4a). Figure 4b presents a schematic representation of a typical force–deformation curve for hardness, showing the peak force that indicates the hardness of the sample.

### 2.5. Functional Properties of Okara Flour and Crispy Waffles

#### 2.5.1. Sample Preparation

Okara flour was prepared as described in 2.1 by drying and milling the okara into a fine powder. The waffle samples were ground into a uniform powder after baking to prepare them for further analysis.

#### 2.5.2. Cholesterol Adsorption Capacity (CAC)

The cholesterol adsorption capacity of samples was determined according to the method described by [14] with slight modification. The 1 mg/mL of cholesterol solution was prepared in ethanol. The pH of the cholesterol solution was adjusted to pH 2.0 and 7.0, to simulate the acidic environment of the stomach and the neutral environment of the intestines, respectively. These pH levels were chosen to reflect the conditions that the waffles might encounter during digestion, allowing for an understanding of how okara flour affects cholesterol adsorption under physiologically relevant conditions. Next, 2.0 g of sample powder was dispersed in 100 mL of cholesterol solution by continuous stirring at 37 °C for 2 h followed by centrifugation at 6000× *g* for 15 min. The supernatant determined the cholesterol content using Total Cholesterol Assay Kit (Colorimetric) (Cell Biolabs, Inc, San Diego, CA, USA). The cholesterol adsorption capacity was calculated as the following equation.

$$\mathrm{CAC}\;(\mathrm{mg}/g)=\frac{Cholesterol\;before\;adsorption\;(mg)-Cholesterol\;after\;adsorption\;(mg)}{Weight\;of\;\mathrm{dry}\;sample\;(g)}$$

#### 2.5.3. Glucose Adsorption Capacity (GAC)

The glucose adsorption capacity of samples was determined according to the method described by [14] with slight modification. Here, 1 g of sample was dispersed in 100 mL of 50 mM glucose by continuous stirring at 37 °C for 6 h followed by centrifugation at 4000× *g* for 20 min. The glucose content in the supernatant was measured by Glucose Oxidase Assay Kit (Fluorometric) (Abcam, Cambridge, UK). The glucose adsorption capacity was calculated as the following equation.

$$\mathrm{GAC}\;(\mathrm{mM}/g)=\frac{Glucose\;before\;adsorption\;(mM)-Glucose\;after\;adsorption\;(mM)}{Weight\;of\;\mathrm{dry}\;sample\;(g)}$$

### 2.6. Sensory Evaluation and Consumer Study of Crispy Waffles

All subjects provided informed consent before participating in the study. The study was conducted following international guidelines for human research protection, and the methodology was approved by the Kasetsart University Research Ethics Committee (COE No. COE65/026). The panelists, aged 18 years or older, were employees of the Institute of Food Research and Product Development, Kasetsart University, Bangkok, Thailand. They had previously been recruited for and were familiar with the Sensory and Consumer Research Unit’s sensory evaluation protocol. Sensory evaluation and the consumer study were conducted in two phases.

First, an affective test was carried out by 30 panelists who were habitual consumers of grain-based snacks using a 9-point hedonic scale (1: dislike extremely to 9: like extremely). The panelists were asked to rate the waffle samples replacing glutinous rice flour with 30% and 40% okara flour on their appearance, flavor, taste, texture, and overall liking to determine the optimum replacement level. These specific substitution levels were selected for sensory evaluation because they represented the most significant changes in the physical properties of the waffles, allowing us to focus on the levels where sensory attributes were most likely to be impacted.

Second, a Check-All-That-Apply (CATA) analysis was conducted by 60 consumers to profile sensory descriptions and product concepts and define the drivers of liking for waffles with 0% and 30% okara flour replacing glutinous rice flour compared against three commercial benchmarks. These benchmarks included commercial product brand 1 with coconut milk flavor (Farm rak brand, Anantra Food and Marketing Limited Partnership, Angthong Thailand) and brands 2 and 3 with mixed fruit and vegetable flavor and mango corn and pumpkin flavor, respectively (Apple monkey brand, Healthy Foods Co., Ltd., LopBuri, Thailand). The questionnaire design followed our previous study [15] with some modifications. For each sample, consumers evaluated overall liking using the 9-point hedonic scale, followed by the open-ended questions “What do you like about the product?” and “What do you dislike about the product?” Consumers were then provided with a CATA list of 9 sensory attributes (Crispy, Hard, Sweet, Salty, Nutty, Coconut, Fruity, Oily, and Dry/Powdery) and eight product positioning phrases (“Made entirely from plants”, “High protein, High in dietary fiber”, “Gluten free, Vegan (egg and dairy free)”, “Long shelf life”, “No added color or scent”, “No preservatives added”). These 17 attributes were selected from the descriptive analysis of similar cellulosic by-products and food systems [6,16,17,18].

Each sample treatment, coded with three random digits, consisted of one piece of waffle (approximately 1.3–1.9 g). They were presented to the panelists in clear, press-seal plastic bags to prevent moisture absorption. Natural water was provided as a taste neutralizer between products. The sensory evaluation and consumer study were conducted as a full crossover, where each panelist evaluated each sample in a sequential monadic presentation. Furthermore, the presentation order of the sample products across panelists was balanced, and the assignment of the presentation order to the panelists was randomized.

### 2.7. Storage Stability of Crispy Waffle

Waffles with a 30% okara flour replacement were sealed in laminated aluminum foil pouches and stored under ambient conditions. These products were divided into three groups for analyses at 0, 30, and 60 days to assess physical and chemical properties, including moisture content, water activity, color, and texture (hardness). Microbiological assessments were conducted at only the 0- and 60-day time points. The microbiological analysis aimed to detect yeast, mold, *Escherichia coli*, *Staphylococcus aureus*, *Clostridium perfringens*, *Bacillus cereus*, *and Salmonella* spp., following the Thai Department of Medical Sciences (DMSC) standards [19]. This methodology aligns with that of Aussanasuwannakul and Butsuwan (2024) [15], which included similar ambient storage conditions at 25 °C and 60% relative humidity, and parallel microbiological quality assessments according to local regulations.

### 2.8. Statistical Analysis

To compare the means of physical properties (width, thickness, spread ratio, water activity, moisture content, color, and hardness), proximate composition, cholesterol adsorption capacity (CAC), and glucose adsorption capacity (GAC) among different treatments of okara waffles, an analysis of variance (ANOVA) was performed using XLSTAT 2023.3.0 (1415) software for Macintosh 14.2 [20]. Post hoc Tukey’s tests were conducted to determine significant differences between means. The results are presented as mean ± standard deviation, with a significance threshold set at *p* ≤ 0.05. All analyses were replicated at least three times to ensure reliability and robustness of the data.

For sensory evaluation data, XLSTAT’s CATA data analysis tool was used to automate the analysis of the Check-All-That-Apply (CATA) data, including Cochran’s Q test and correspondence analysis (CA). Penalty-lift analysis was conducted to understand the impact of various sensory attributes on overall acceptability scores. The sensory evaluation results were visualized using correspondence analysis biplots, penalty-lift bar graphs, and principal coordinate analysis (PCoA) maps to illustrate the relationships between sensory attributes, product positioning, and consumer liking.

Independent *t*-tests were performed to compare sensory scores (appearance, soybean flavor, taste, texture, and overall liking) between the 30% and 40% okara flour replacement groups. To analyze the percentage acceptance and non-acceptance data, a Chi-square test was utilized to compare the proportions across the two groups, testing the hypothesis that the proportions of acceptance and non-acceptance are equal between the treatments.

For the rheological properties, one-way ANOVA was used to compare means across the four groups (0%, 30%, 40% okara flour with 60 and 80 mesh), followed by Tukey’s HSD test for pairwise comparisons. Independent *t*-tests were used for two-group comparisons (e.g., 30% vs. 40% okara flour or 60 mesh vs. 80 mesh).

ANOVA was employed to compare the quality of waffle samples over the 0, 30, and 60-day storage periods, allowing for the identification of overall differences among group means. Post hoc Tukey’s tests were used to determine which specific groups differed significantly.

## 3. Results

### 3.1. Viscoelastic Properties of Waffle Batters

The rheological properties of waffle batter were analyzed under two treatments: okara flour replacement levels (30% and 40%) and particle size ranges (60 mesh and 80 mesh). The results from the oscillatory shear tests and creep-recovery tests are presented in Table 2.

The amplitude-sweep results revealed significant differences in the flow point and the limit of the linear viscoelastic region (LVR) among the samples. The control batter (0% okara flour) exhibited the highest flow point at 20.34%, indicating a higher resistance to flow compared to the batter with okara flour. The batter with 30% okara flour (60 mesh) showed a substantial decrease in the flow point to 2.17%, while the batter with 40% okara flour (60 mesh) had a flow point of 3.76%. The 40% okara flour batter with 80-mesh particle size displayed a higher flow point of 4.70% compared to the 60-mesh particle size at the same replacement level. In terms of the limit LVR, the control batter had the lowest value at 0.02%. In contrast, the batters with 30% and 40% okara flour (60 mesh) showed higher limit LVR values of 0.06% and 0.08%, respectively, with the 40% okara flour batter (80 mesh) exhibiting the highest limit LVR at 0.09%.

The frequency-sweep tests provided insights into the loss factor (tanδ) and complex viscosity (η*). The control batter had a tanδ of 0.45, indicating a higher viscous behavior. In contrast, the batters with okara flour substitution exhibited lower tanδ values, with the 30% replacement (60 mesh) at 0.21, and the 40% replacement (60 mesh and 80 mesh) at 0.20 and 0.18, respectively. The complex viscosity of the control batter was 3.94 Pa·s. For the batters with okara flour, the complex viscosity increased significantly, with the 30% okara flour batter (60 mesh) at 5862 Pa·s, and the 40% okara flour batters showing values of 5539.30 Pa·s (60 mesh) and 10,923.33 Pa·s (80 mesh). This indicates a pronounced effect of both okara flour content and particle size on viscosity.

The creep and recovery tests provided further insights into the viscoelastic behavior of the batters. Elastic recovery ($\gamma_{e}$) was only observed in batters containing okara flour. The 30% okara flour batter (60 mesh) had a $\gamma_{e}$ of 0.06%, while the 40% okara flour batters (60 mesh and 80 mesh) exhibited $\gamma_{e}\;$ values of 0.05%. Viscous loss ($\gamma_{v}$) in the 30% okara flour batter (60 mesh) was 0.12%, compared to 0.09% and 0.05% for the 40% okara flour batters (60 mesh and 80 mesh, respectively). The maximum deformation ($\gamma_{max}$) was higher in the 30% okara flour batter (0.19%) than in the 40% okara flour batters (0.14% for 60 mesh and 0.10% for 80 mesh). Zero-shear viscosity ($\eta_{0}$) values were similar across all okara flour batters, ranging from 6.91 × 10^9^ to 1.71 × 10^10^ mPa·s. Shear compliance values varied slightly, with the 30% okara flour batter (60 mesh) showing 6.47 × 10^−5^ 1/Pa, and the 40% okara flour batters had values of 1.38 × 10^−4^ (60 mesh) and 9.99 × 10^−5^ (80 mesh) 1/Pa.

**Figure 5:**
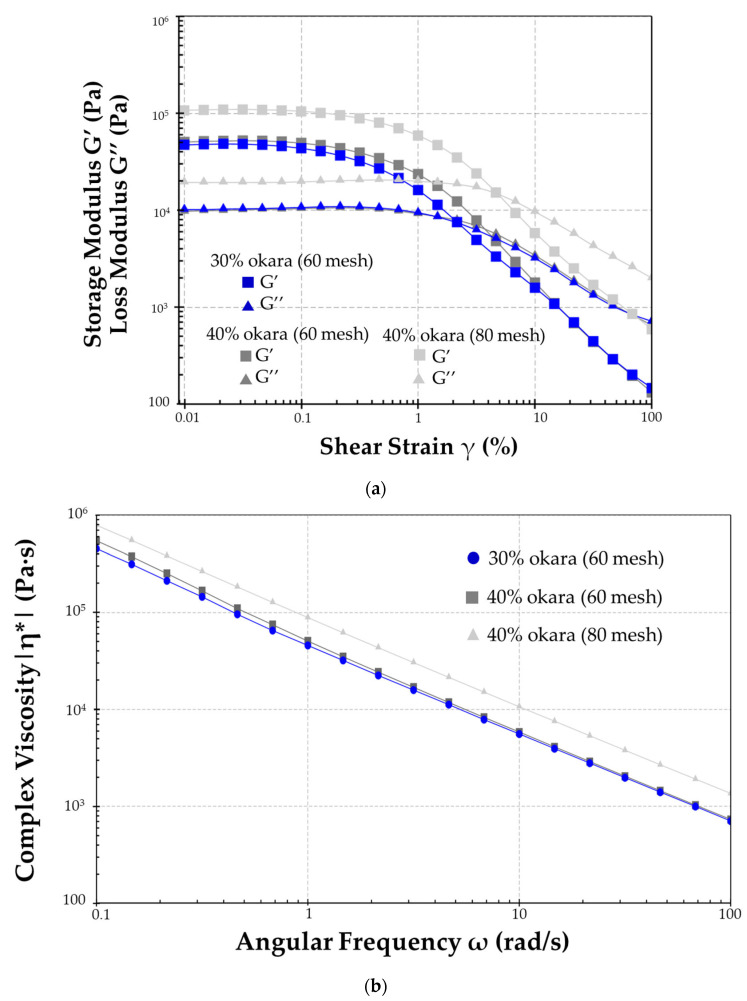

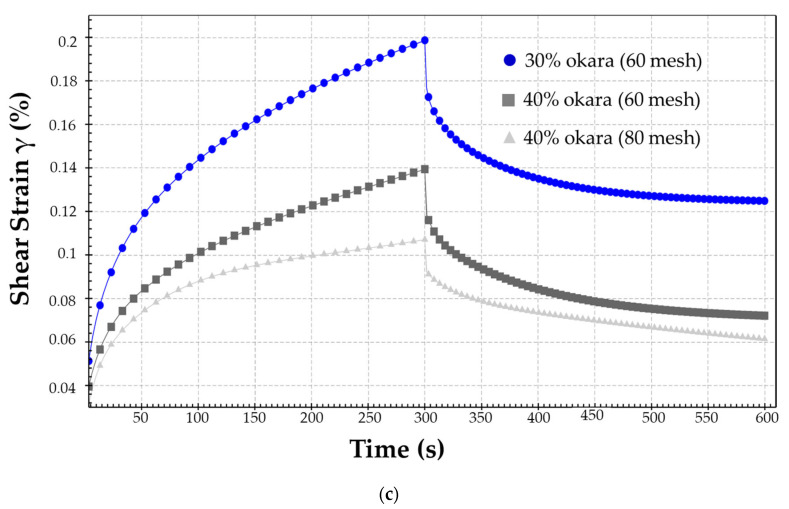
(**a**) Storage modulus (G′) and loss modulus (G″) of waffle batters with varying okara flour content and particle sizes during amplitude-sweep tests. (**b**) Complex viscosity as a function of frequency during frequency-sweep tests. (**c**) Shear strain over time for waffle batters with varying okara flour content and particle sizes during creep–recovery tests.

Figure 5a,b illustrate the storage modulus (G′) and loss modulus (G″) as well as the shear strain over time during the amplitude sweep and creep and recovery tests, respectively. These figures further highlight the differences in rheological properties between the batters with varying okara flour content and particle sizes.

### 3.2. Physical Properties and Proximate Composition of Crispy Waffles

#### 3.2.1. Spread Ratio, Moisture Content, Water Activity, and Surface Color

Substituting glutinous rice flour with okara flour in crispy waffles was evaluated at 10%, 20%, 30%, and 40% levels. The physical properties analyzed included spread ratio, moisture content, water activity, and color. The spread ratio of okara waffles decreased with increasing okara flour content, with the 10% okara waffles having the highest spread ratio (381.11 ± 42.39), while the 40% okara waffles had the lowest (182.28 ± 40.43) (Table 3). Moisture content and water activity also showed significant increases with higher okara flour substitution. For instance, the moisture content increased from 4.0754% ± 0.0251% in the 30% okara waffles to 4.6989% ± 0.0175% in the 40% okara waffles, and water activity followed a similar trend. Color analysis revealed a significant decrease in brightness (L*) and yellowness (b*) values with more okara flour. The 10% okara waffles had a brightness (L*) of 62.32 ± 0.76, which decreased to 53.78 ± 0.86 in the 40% okara waffles. Similarly, the yellowness (b*) decreased from 13.82 ± 0.33 to 17.28 ± 0.04, while the redness (a*) values did not differ significantly. Hardness increased significantly with more okara flour. The 10% okara waffles had a hardness of 174.97 ± 16.22 g/s, whereas the 40% okara waffles had a hardness of 307.65 ± 20.86 g/s. However, the hardness values for 20% to 40% okara waffles were not significantly different from each other.

**Figure 6:**
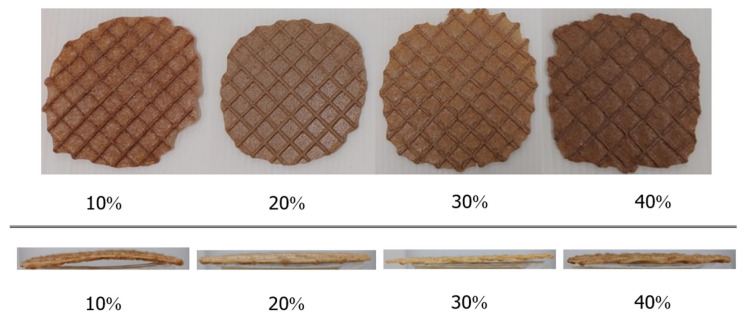
Top view and side view of okara waffles, cocoa flavor, with okara flour content of 10%, 20%, 30%, and 40%, respectively.

As illustrated in Figure 6, the top view and side view of crispy waffles with varying okara flour content (10%, 20%, 30%, and 40%) show noticeable changes in physical appearance and texture. The visual differences highlight how okara flour affects the spread ratio and texture of the waffles.

#### 3.2.2. Nutritional Composition

The nutritional composition of okara flour and waffles (30% okara flour substitution) was analyzed, revealing that okara flour is rich in protein (35.47%) and carbohydrates (54.8%). In contrast, the waffles had a higher fat content (19.12%) and lower protein content (10.77%) (Table 4).

### 3.3. Cholesterol and Glucose Adsorption Capacities

The cholesterol adsorption capacity (CAC) and glucose adsorption capacity (GAC) of okara flour, as well as waffles with 0% and 30% okara flour substitution, were evaluated at pH 2 and pH 7. The results are summarized in Table 5. These capacities were calculated based on the measured concentrations of cholesterol and glucose in the supernatant, using colorimetric and fluorometric kits, respectively. Waffles with 0% and 30% okara flour were selected for these evaluations because the 30% substitution level was preferred in the sensory analysis and represented an extreme of the substitution levels studied, allowing us to highlight the most significant differences in functional properties.

At pH 2, the CAC of okara flour was 366.24 ± 3.03 mg/g, while at pH 7, it was 313.84 ± 23.34 mg/g. For the 0% okara flour waffle sample, the CAC values were 639.24 ± 7.90 mg/g at pH 2 and 641.04 ± 5.28 mg/g at pH 7. The 30% okara flour waffle sample showed CAC values of 640.07 ± 2.83 mg/g at pH 2 and 618.71 ± 6.60 mg/g at pH 7.

The GAC of okara flour was 99.27 ± 0.27 mM/g. In comparison, the GAC values for the 0% and 30% okara flour waffle samples were 182.72 ± 0.74 mM/g and 183.82 ± 0.54 mM/g, respectively.

The data indicate that the incorporation of okara flour into waffle formulations did not significantly affect the CAC at pH 2 or the GAC. However, the CAC at pH 7 decreased with the 30% okara flour substitution compared to the control (0% okara flour). These findings suggest that while okara flour itself has high CAC and GAC values, its incorporation into waffles does not significantly enhance these properties.

### 3.4. Storage Stability

The waffles with 30% okara flour were evaluated for storage stability over 60 days. The storage conditions included assessments of moisture content, water activity, color (L*, a*, b* values), hardness, and microbiological quality. Table 6 provides a detailed summary of the storage stability results for these parameters.

The moisture content and water activity remained within acceptable ranges throughout the 60-day period. Specifically, the moisture content increased from 4.640% ± 0.029% at day 0 to 4.932% ± 0.009% at day 60, indicating a significant change (*p* < 0.05). Water activity showed a slight fluctuation, starting at 0.378 ± 0.004 on day 0, decreasing to 0.354 ± 0.004 on day 30, and then increasing again to 0.371 ± 0.003 by day 60, with significant differences noted between the measurements at each time point.

The color measurements indicated slight variations over time. The brightness (L*) value showed a significant increase from 60.36 ± 1.34 at day 0 to 61.38 ± 1.44 at day 60, while the redness (a*) value decreased from 9.76 ± 0.32 to 9.27 ± 0.11, and the yellowness (b*) value remained relatively stable at around 15.46 ± 0.57 to 15.38 ± 0.18.

The hardness of the waffles decreased significantly over the storage period. Initially, the hardness was 363.80 ± 11.47 g/sec, which decreased to 218.88 ± 10.45 g/sec at day 30 and, further, to 211.42 ± 6.09 g/sec at day 60, highlighting a substantial reduction in texture firmness.

Microbiological assessments showed that the levels of yeasts, molds, *E. coli*, *C. perfringens*, *B. cereus*, and *S. aureus* were within safe limits throughout the storage period. Yeasts were detected at <10 CFU/g at both day 0 and day 60, while molds were detected at <10 CFU/g on day 0 and 60 CFU/g at day 60. *E. coli* remained at <3 MPN/g, and *C. perfringens*, *B. cereus*, and *S. aureus* were all maintained at levels <10 CFU/g or <100 CFU/g for *B. cereus* throughout the study. *Salmonella* spp. were not detected (ND) on either day, indicating excellent microbiological stability of the product.

### 3.5. Sensory Evaluation and Consumer Study

#### 3.5.1. Affective Test

Waffles with 30% and 40% okara flour were evaluated by 30 panelists to determine the optimal substitution level based on sensory attributes. The sensory evaluation results are presented in Table 7.

The results indicated that there were no significant differences between the 30% and 40% okara waffles in terms of appearance, soybean flavor, and overall flavor. However, the 30% okara waffles received higher scores for texture and overall liking, with mean values of 7.37 ± 1.50 and 7.43 ± 1.41, respectively. In contrast, the 40% okara waffles had lower scores for these attributes, with mean values of 6.53 ± 1.74 for texture and 6.50 ± 1.57 for overall liking. Additionally, 87% of panelists accepted the 30% okara waffles, whereas only 80% accepted the 40% okara waffles. This suggests that 30% okara flour is the optimal level for use in crispy waffles.

#### 3.5.2. Check-All-That-Apply (CATA) Analysis

Cochran’s Q Test

The Cochran’s Q test results presented in Table 8 reveal significant differences among the sensory attributes evaluated in the waffle samples, indicating distinct sensory profiles for each variant.

For the “Okara 0%” sample, consumers highlighted attributes such as “crispy” (0.980), “salty” (0.640), “coconut” (0.760), and “oily” (0.520) as significantly prominent compared to the other samples. These high values suggest that “Okara 0%” waffles are perceived as having a crispy texture, a salty taste, a notable coconut flavor, and an oily mouthfeel.

In contrast, the “Okara 30%” sample was characterized as being “hard” (0.700), “nutty” (0.640), and “dry/powdery” (0.220). Additionally, “Okara 30%” stood out for its nutritional attributes, being closely associated with “high protein” (0.560), “high fiber” (0.400), and “gluten-free” (0.480). However, for each of the samples, an attribute independence test determined that the attributes “high protein” and “high fiber” are redundant for the “Okara 30%” sample. The Bonferroni-corrected significance level was 0.00075758, indicating these attributes are not independent.

Among the commercial benchmarks, “Benchmark 1” was frequently described as “sweet” (0.760) and “gluten-free” (0.500), highlighting its appeal in terms of taste and dietary benefits. “Benchmark 2” was associated with “fruity” (0.800) and “crispy” (0.920) attributes, reflecting its favorable sensory qualities. Meanwhile, “Benchmark 3” showed a notable relationship with the “fruity” attribute (0.400), although it was less distinctly associated with other specific attributes.

Overall, both “Okara 0%” and “Okara 30%” were relatively far from “Benchmark 2” and “Benchmark 3” in terms of sensory attributes, underscoring their unique profiles. These findings demonstrate the significant impact of okara flour substitution on the sensory and nutritional characteristics of waffles, providing valuable insights for product development to enhance consumer satisfaction.

Correspondence Analysis (CA)

**Figure 7:**
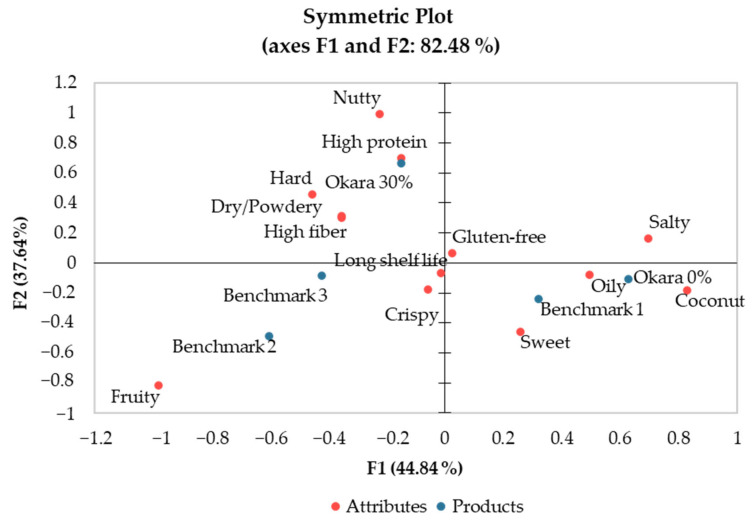
Biplot from correspondence analysis (CA) exhibiting the relationship between CATA attributes and waffle samples.

Correspondence analysis (CA) was employed to explore how consumers described the waffle samples using the Check-All-That-Apply (CATA) attributes. This method allows us to visualize the selection frequency data on a map, illustrating the relationships between samples and their associated sensory attributes. The first axis captured 44.84% of the total variability, while the second axis accounted for 37.64%, resulting in a cumulative 82.48% of the variability captured by the first two axes (Figure 7).

The CA biplot shows the positioning of the waffle samples relative to their sensory attributes. The “Okara 30%” sample is prominently associated with attributes such as “nutty”, “hard”, and “dry/powdery”. These attributes highlight the textural and flavor characteristics imparted by the higher okara flour content. Additionally, “Okara 30%” is strongly linked with “high protein” and “high fiber” product positioning, emphasizing its nutritional benefits.

In contrast, the “Okara 0%” sample is closely associated with “oily”, “salty”, and “coconut” attributes, indicating these were the key sensory characteristics for this variant. The “Okara 0%” sample also aligns closely with “Benchmark 1”, suggesting some similarities in sensory profiles between these two products.

Both “Okara 0%” and “Okara 30%” are relatively distant from “Benchmark 2” and “Benchmark 3”, which underscores their unique sensory profiles. “Benchmark 2” is characterized by attributes like “fruity” and “crispy”, reflecting its appealing sensory qualities. Meanwhile, “Benchmark 3” shows a notable relationship with the “fruity” attribute but does not prominently feature other specific attributes.

This analysis provides a clear depiction of how the different waffle samples are perceived in terms of their sensory attributes, offering valuable insights for product development and marketing strategies aimed at enhancing consumer acceptance and satisfaction.

Penalty-lift Analysis

**Figure 8:**
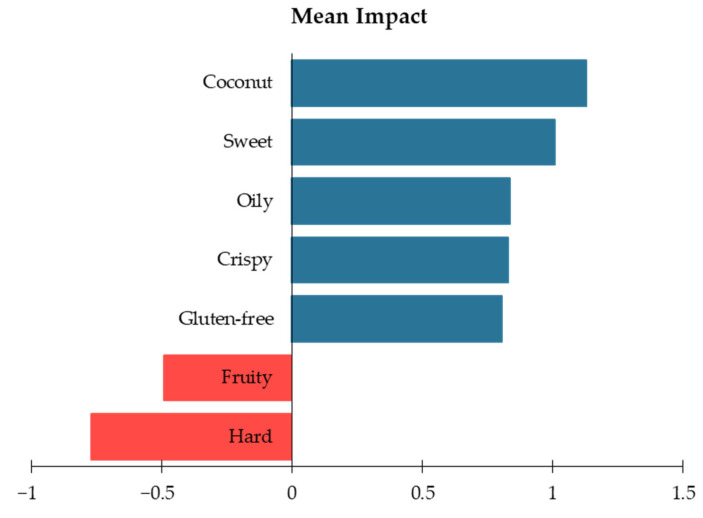
Bar graph from penalty-lift analysis showing the mean impact of sensory attributes on consumer liking of waffle samples.

Figure 8 provides a detailed look into the mean impact of various sensory attributes on the overall acceptability scores of the waffle samples, as measured on a 9-point hedonic scale. The penalty-lift analysis identifies attributes that significantly influence consumer liking, either positively or negatively. Attributes such as “coconut” (mean impact 1.13), “sweet” (1.00), “oily” (0.83), and “crispy” (0.82) were found to have significant positive impacts on the overall liking scores (*p* < 0.05). These attributes are critical for enhancing the appeal of the waffle products. Conversely, attributes like “hard” (−0.77) and “fruity” (−0.49) had significant negative impacts on consumer liking. These findings highlight the importance of specific sensory attributes in driving consumer preference, suggesting that formulations should emphasize positive attributes while minimizing negative ones to improve market acceptance. These trends are reflected in Figure 7 as well.

Principal Coordinate Analysis (PCoA)

**Figure 9:**
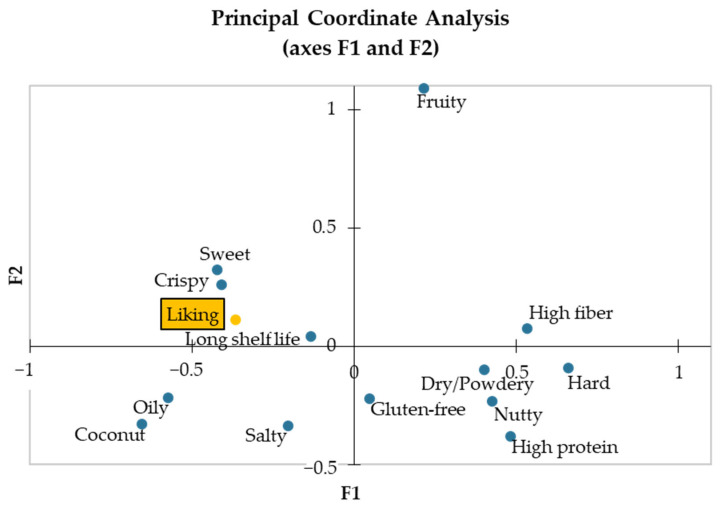
Two-dimensional map from principal coordinate analysis (PCoA) depicting the interplay between sensory attributes, product positioning, and consumer liking.

Principal coordinate analysis (PCoA) was conducted to further explore the relationships between sensory attributes, product positioning, and consumer liking. As shown in Figure 9, the PCoA plot visualizes these relationships in a two-dimensional space, capturing 23.49% of the variability on the first axis (F1) and 17.07% on the second axis (F2), cumulatively explaining 40.56% of the variability.

The PCoA map reveals that consumer liking is closely associated with attributes such as “sweet” and “crispy”, along with the product positioning “long shelf life”. These attributes and positioning significantly drive consumer preference, as indicated by their proximity to the liking vector in the plot. The analysis shows that waffle samples exhibiting these positive attributes are more likely to be preferred by consumers.

The visualization in Figure 9 also helps identify how different waffle samples are perceived based on their sensory profiles and nutritional claims. This detailed understanding of consumer preferences provides valuable insights for refining product formulations and marketing strategies to align with consumer tastes and enhance overall satisfaction.

## 4. Discussion

The introduction of okara in gluten-free products, like our crispy waffle, poses challenges due to the absence of gluten’s viscoelastic properties (Brites et al., 2018; Demirkesen and Ozkaya, 2022) [21,22]. Gluten-free products often face issues related to texture, taste, and structure, which are amplified by the lack of gluten’s binding capabilities (Egea et al., 2023) [23]. The high fiber content in okara, while nutritionally commendable, can influence the waffle’s texture and moisture retention. Additionally, the protein in okara might affect Maillard reactions during baking, which can alter the color and flavor profiles of the waffles (Brites et al., 2018) [21]. Achieving a balance between the desired crispy texture and the nutritional benefits of okara is, thus, crucial. The use of okara in bakery items can modify their sensory properties, potentially introducing a beany flavor, which might affect consumer acceptability (Guimarães et al., 2019; Park et al., 2015) [24,25]. The fiber and protein content in okara can significantly influence the texture and crispness of the waffle. The high moisture content in okara can lead to a softer interior, potentially affecting the desired crispy exterior. While both are dried products, in the context of extruded snacks, the interior texture might be more of a concern (Aussanasuwannakul et al., 2022) [6].

Impact of Okara Flour Content:

Increasing the okara flour content in the batter significantly affected its rheological properties. The decrease in the flow point with higher okara flour content suggests that the batter becomes more solid-like and more resistant to flow (Aussanasuwannakul et al., 2020) [26]. This is likely due to the high fiber content of okara flour, which disrupts the network structure of the batter, enhancing its rigidity under stress. Additionally, the increase in complex viscosity (η*) with higher okara flour content indicates that the batter exhibits greater resistance to deformation, which could impact the ease of handling and processing. These findings align with those of Olakanmi et al. (2022) [27], who reported that the incorporation of dietary fibers like okara significantly increased dough viscosity and elasticity, enhancing both the nutritional profile and textural properties of bakery products.

Effect of Particle Size:

The particle size of okara flour also played a crucial role in determining the rheological behavior of the batter. Batters with smaller particle sizes (80 mesh) exhibited higher flow points and complex viscosities compared to those with larger particle sizes (60 mesh). This suggests that finer okara particles create a denser and more cohesive network within the batter, enhancing its structural integrity. However, this also means that the batter may be more difficult to mix and spread evenly during cooking, potentially affecting the texture and appearance of the final product. This observation is supported by Aussanasuwannakul et al. (2023) [7], who found that finer particle sizes of okara flour provided additional rigidity and enhanced interaction with liquid components, leading to improved dough cohesiveness and stability.

Viscoelastic Properties:

The loss factor (tanδ) values indicated that batters with okara flour have lower viscous behavior compared to the control batter. This aligns with the expectation that okara flour, being rich in dietary fiber, contributes to a more elastic and less flowable batter. The amplitude-sweep results, showing higher limit LVR values for batters with okara flour, further support this observation, indicating that these batters can withstand greater strain before deviating from their linear viscoelastic region. Similar results were reported by Cappelli et al. (2020) [28], who demonstrated that different flour types and ingredients significantly impact dough elasticity and viscosity, with certain blends enhancing dough stability and texture.

Creep–Recovery Behavior:

The creep–recovery tests provided additional insights into the viscoelastic nature of the batters. The elastic recovery ($\gamma_{e}$) and viscous loss ($\gamma_{v}$) values demonstrated that batters with okara flour exhibited viscoelastic behavior, with no creep-recovery behavior measured in the control batter. This suggests that okara flour enhances the elastic properties of the batter, which is beneficial for maintaining the desired texture of the waffles after cooking. It was found that the control batter did not exhibit creep and recovery behaviors as the rheometer could not detect or measure them within the range of shear strain studied.

Focusing on the effect of okara flour replacement levels, the $\gamma_{max}$ value for the 30% okara flour batter was higher than that for the 40% okara flour batter (0.19% vs. 0.14%, respectively). This indicates that the batter with a lower percentage of okara flour (30%) experienced greater maximum deformation. Additionally, the $\gamma_{v}$ value for the 30% okara flour batter was higher (0.12%) compared to the 40% okara flour batter (0.09%), suggesting more permanent deformation occurred in the batter with lower okara content. However, the differences in $\gamma_{e}$ values between the 30% and 40% okara flour batters were not significant, indicating that both levels of okara flour similarly enhanced the elastic recovery of the batter.

For both the 60-mesh and 80-mesh samples, the $\gamma_{max}$ determined by the creep tests is higher than the limiting LVE range ($\gamma_{L}$, 0.07–0.09%) suggested by the amplitude sweep test, indicating that irreversible structural changes occurred during the creep and recovery test. However, the test was unable to significantly discriminate between the particle sizes regarding viscoelastic behavior. The lower shear compliance J(t) value in the 80-mesh sample might suggest higher rigidity than the 60-mesh sample, but the difference was not statistically significant (9.99 × 10^−5^ vs. 1.38 × 10^−4^; *p* > 0.05). Tridtitanakiat et al. (2023) [17] found similar improvements in the viscoelastic properties of gluten-free rolls with okara flour, reporting significant increases in storage modulus (G′) and loss modulus (G″) and improved structural stability as evidenced by creep–recovery tests.

Physical Properties and Proximate Composition of Crispy Waffles:

The physical properties of okara waffles were also significantly influenced by the substitution levels of okara flour. As the okara flour content increased, the spread ratio of the waffles decreased, with the 10% okara waffles having the highest spread ratio and the 40% okara waffles having the lowest. This is indicative of the increasing viscosity and reduced flowability of the batter as more okara flour is added. 

The observed pattern, where values for certain physical properties initially increase at 10% okara flour, then decrease, and increase again at 40%, can be attributed to the complex interactions between the okara flour, moisture content, and the structural integrity of the batter. At 10% substitution, the added okara flour likely enhances the water-binding capacity, leading to improved texture and stability, which is reflected in higher values for properties like hardness. However, as the substitution level increases to 20% and 30%, the high fiber content of okara may start to interfere with the batter’s network, reducing its ability to maintain these properties, hence the observed decrease. At 40%, the batter reaches a threshold where the density and rigidity provided by the okara flour’s high fiber content become dominant, leading to a final increase in these physical properties.

The moisture content and water activity also showed significant increases with higher okara flour substitution, which could impact the shelf life and textural properties of the final product. The color analysis revealed a decrease in brightness (L*) and yellowness (b*) with more okara flour, while the hardness of the waffles increased significantly. This aligns with the expectation that higher fiber content and water absorption lead to a denser and firmer product, particularly at higher substitution levels, as noted by Asghar et al. (2023) [29], who found that the addition of okara improved the water-binding capacity and fibrous nature of the dough, leading to enhanced texture and stability.

The proximate composition analysis revealed that okara flour is rich in protein and carbohydrates, while the waffles had a higher fat content and lower protein content after substitution. This is consistent with Chen et al. (2024) [30], who found that the addition of dietary fibers such as insoluble soybean fiber enhanced the nutritional and functional properties of food products.

Cholesterol and Glucose Adsorption Capacities:

The cholesterol and glucose adsorption capacities of okara flour and okara waffles were evaluated at different pH levels to simulate the varying conditions within the human gastrointestinal tract and provide insights into the potential physiological implications of dietary fibers like okara flour.

At pH 2, simulating stomach conditions, okara flour demonstrated high CAC, suggesting effective cholesterol sequestration in an acidic environment. This indicates that dietary fibers with high CAC at pH 2 can bind cholesterol, reducing its availability for absorption in the intestines, potentially lowering overall cholesterol absorption into the bloodstream (Feng et al., 2023; Lyu et al., 2021) [31,32]. Additionally, dietary fibers that bind cholesterol effectively at this pH can also interact with bile acids, promoting their excretion and forcing the body to use more cholesterol for bile acid production, thus lowering circulating cholesterol levels (Liang et al., 2023) [33].

At pH 7, simulating intestinal conditions, the high CAC of okara flour indicates continued cholesterol binding and prevention of absorption through the intestinal walls, contributing to the excretion of cholesterol-bound fibers through feces. This disruption of enterohepatic circulation can further enhance cholesterol excretion and promote cardiovascular health by reducing serum cholesterol levels (Tian et al., 2023; Zhang et al., 2023) [34,35].

The significantly higher cholesterol adsorption capacity (CAC) and glucose adsorption capacity (GAC) observed in the waffles can be attributed to several factors, including the role of ingredients, chemical interactions, hydration, gel matrix formation, binding sites, and surface area and porosity. Glutinous rice flour contributes significantly to the overall texture and adsorption properties of the waffle matrix. Its high amylopectin content leads to gelatinization during baking, creating a gel-like structure that enhances the binding of cholesterol and glucose molecules. The gelatinized starch increases the surface area and provides numerous binding sites for these molecules (Ma et al., 2023) [36]. Coconut milk, with its high fat content, acts as an emulsifier and enhances the structural integrity of the waffle matrix. The fats in coconut milk help to form a stable emulsion, which can trap cholesterol and glucose molecules more effectively. 

The hydration process during the preparation of the waffle batter is crucial for enhancing the functional properties of okara flour. Hydration allows the dietary fibers to swell, increasing their surface area and porosity. This swelling effect exposes more binding sites for cholesterol and glucose adsorption (Hu et al., 2022) [37]. Additionally, the hydration and subsequent baking process lead to the formation of a gel matrix, primarily due to the gelatinization of glutinous rice flour. This gel matrix provides a physical barrier that traps cholesterol and glucose, thereby enhancing their adsorption. The gel-like structure increases the viscosity of the batter, further improving the binding and retention of these molecules (Ma et al., 2023) [36].

The lack of significant differences in CAC at pH 2 and GAC between waffles with 0% and 30% okara flour substitution could be due to the saturation of binding sites in the waffle matrix, suggesting that beyond a certain threshold, additional okara flour does not further increase adsorption capacity. However, the slightly higher CAC at pH 7 in waffles without okara flour might be due to the structural changes in the matrix that facilitate better cholesterol binding at neutral pH, as supported by Ma et al. (2023) [36] and Hu et al. (2022) [37].

Storage Stability:

The storage stability of okara waffles with 30% okara flour was assessed over 60 days, focusing on moisture content, water activity, color, hardness, and microbiological quality. The moisture content exhibited a slight increase, from 4.640% at day 0 to 4.932% at day 60, suggesting a minor redistribution of water within the product over time. This slight rise in moisture content could be linked to the hygroscopic nature of the fiber in okara, which tends to absorb moisture from the environment (Aussanasuwannakul and Butsuwan, 2024) [15].

The water activity (a_w_) showed fluctuations but remained within acceptable limits for preventing microbial growth. It decreased initially from 0.378 at day 0 to 0.354 at day 30, then increased to 0.371 at day 60. These variations indicate that while the product’s ability to support microbial growth might have changed slightly, it remained within safe levels throughout the storage period.

The color measurements (L*, a*, b*) revealed slight changes. Brightness (L*) increased from 60.36 to 61.38, while redness (a*) decreased from 9.76 to 9.27, and yellowness (b*) remained relatively stable. These color changes might be attributed to Maillard reactions and other chemical processes occurring during storage, affecting the visual appeal of the waffles.

The hardness of the waffles significantly decreased from 363.80 g/sec at day 0 to 211.42 g/sec at day 60, indicating a substantial reduction in texture firmness. This reduction in hardness could be due to moisture redistribution and starch retrogradation, leading to a softer texture over time (Aussanasuwannakul and Butsuwan, 2024) [15].

Microbiological assessments confirmed the safety of the waffles over the storage period. Yeasts were consistently detected at <10 CFU/g, and molds were detected at 60 CFU/g on both day 0 and day 60, while molds were detected at <10 CFU/g on day 0 and increased to 60 CFU/g at day 60. Pathogens such as *E. coli*, *C. perfringens*, *B. cereus*, and *S. aureus* remained below detectable levels (<10 CFU/g or <100 CFU/g for *B. cereus*) or were not present, indicating good microbiological stability and compliance with food safety standards.

Overall, the storage stability evaluation suggests that okara waffles with 30% okara flour maintain acceptable moisture content, water activity, color, and microbiological safety over a 60-day period. However, the significant reduction in hardness highlights the need for formulation adjustments to maintain textural quality during extended storage.

Sensory Evaluation and Consumer Study:
Impact of Okara Flour on Sensory Attributes

The incorporation of okara flour into food products offers significant nutritional benefits but also presents challenges in sensory attributes. Kreger et al. (2012) [38] demonstrated in their study that different protein types (whey and soy) and levels significantly influence sensory characteristics such as toasted corn aroma-by-mouth, roasted soy aroma-by-mouth, puffed and porous appearance, crispiness, buttery aroma-by-mouth, sweetness, and crunchiness in high-protein extruded snacks. Similarly, Lee et al. (2020) [39] found that higher okara substitution levels of 20% and 40% in biscuits improved nutritional quality but led to increased hardness and undesirable beany flavors. This aligns with our findings, where the “Okara 30%” waffle exhibited increased hardness and a led to a dry/powdery texture, negatively impacting its overall liking. The studies collectively highlight that the chemical composition of okara, particularly its high fiber and protein content, plays a crucial role in these sensory changes, indicating the need for careful formulation adjustments to balance nutrition and sensory appeal.

Enhancing Texture in Okara-Based Crispy Snacks

Enhancing the texture of okara-based products is essential for improving consumer acceptance. Liu et al. (2021) [40] showed that modifying okara fibers through ultrasonic and high-speed shearing treatment improved dough rheology and textural properties. Specifically, they reported that the modified okara fiber increased the water-holding capacity (WHC) and oil-holding capacity (OHC), which, in turn, increased the apparent viscosity, storage modulus (G′), and loss modulus (G″) of the dough. These rheological parameters indicated a more stable and elastic dough structure. In terms of textural properties, the modified okara fiber increased the hardness and gumminess of the cookie dough. Aussanasuwannakul et al. (2022) [6] found that incorporating okara at levels up to 40% in extruded snacks decreased crispness and increased hardness. The combination of ingredients in their extruded snacks included okara, mung bean flour, and rice flour. These insights suggest that employing similar modifications and processing techniques, such as fermentation and optimized ingredient ratios, could enhance the texture of the “Okara 30%” waffle, achieving a balance between crispiness and reduced hardness.

Preference Mapping and Sensory Optimization

Understanding consumer preferences and optimizing sensory attributes are crucial for product development. The principal coordinate analysis (PCoA) in our study captured 40.56% of the variance, with the first axis (F1) explaining 23.49% and the second axis (F2) explaining 17.07%. Oliveira et al. (2023) [16] used PCA to analyze the sensory attributes of almond skin-supplemented waffles, explaining 82.48% of the variance in their dataset. They highlighted attributes like “colour”, “roasted aroma”, and “dissolubility” as important for consumer preference. In our study, attributes such as “sweet”, “crispy”, and “long shelf life” significantly drive consumer liking. Comparing the two studies, while both identified texture and flavor attributes as critical, our study emphasized sweetness and crispiness more, likely due to the different sensory profiles imparted by okara versus almond skin.

Ingredient Combinations and Quality Enhancement

Zhao et al. (2024) [41] studied the impact of different protein powders—soybean protein isolate (SPI), ovalbumin (OVA), and whey protein (WP)—on the quality of gluten-free pasta. Their study focused on sensory quality, examining how these ingredients affected cooking characteristics and texture. They found that higher concentrations of SPI increased cooking loss, while OVA and WP improved gelatinization, gel stability, and textural properties like chewiness, hardness, elasticity, and tensile strength. This highlights the importance of selecting the right protein type and concentration to optimize sensory quality. In our context, a similar careful selection of ingredients and their combinations can enhance the sensory attributes of okara-based waffles, ensuring they meet consumer preferences for texture and flavor.

By integrating these insights, we can develop strategies to improve the sensory appeal of the “Okara 30%” waffle. Enhancing texture through fermentation, optimizing ingredient ratios, and understanding consumer preferences will lead to a more appealing and nutritionally beneficial product. This not only promotes food waste valorization but also meets consumer demands for healthier and enjoyable snack options.

Future Research Directions:

Future research should focus on optimizing the formulation of okara-based waffles to enhance their sensory attributes while maintaining their nutritional benefits. Testing different ingredient combinations and processing methods, as demonstrated by the studies reviewed, can help achieve a better balance of texture and flavor. Additionally, expanding consumer testing to a broader demographic will provide deeper insights into preferences and help tailor products to meet diverse consumer needs.

## 5. Conclusions

Okara flour proves to be a valuable ingredient for enhancing the nutritional profile of gluten-free crispy waffles by increasing dietary fiber and protein content. This study highlighted the challenges and benefits associated with incorporating okara flour, including its impact on batter viscosity, elasticity, spread ratio, and texture. While higher levels of okara flour improved the nutritional content, they also resulted in increased hardness and altered color, affecting the overall sensory appeal. The optimal substitution level of 30% okara flour was identified, offering a balance between improved nutrition and acceptable sensory qualities. Storage stability tests confirmed that waffles with 30% okara flour maintained good moisture content, water activity, and microbiological safety over 60 days, despite a reduction in hardness. Future research should focus on optimizing ingredient combinations and processing techniques to further improve the sensory and functional properties of okara-based products, ensuring their appeal to consumers and supporting sustainable food production practices. This study underscores the potential of okara flour in promoting food waste valorization and developing nutritious, appealing gluten-free food options.

## Figures and Tables

**Table 1:** Composition of waffle batter with varying percentages of okara flour substitution.

Ingredients	Okara Flour (%)				
	0	10	20	30	40
Okara flour (g)	0.0	3.5	7.0	10.5	14.0
Glutinous rice flour (g)	35.0	31.5	28.0	24.5	21.0
Tapioca flour (g)	4.0	4.0	4.0	4.0	4.0
Coconut milk (g)	53.0	53.0	53.0	53.0	53.0
Cocoa powder (g)	1.0	1.0	1.0	1.0	1.0
Bakery sugar (g)	6.1	6.1	6.1	6.1	6.1
Salt (g)	0.8	0.8	0.8	0.8	0.8
Baking powder (g)	0.1	0.1	0.1	0.1	0.1

**Table 2:** Oscillatory shear and creep-recovery analyses of okara waffles batter across different okara flour replacement levels (0%, 30%, and 40%) and particle sizes (60 and 80 mesh).

		Okara Flour Replacement/Particle Size			
Analyses	Parameters	0% 60 Mesh	30% 60 Mesh	40% 60 Mesh	40% 80 Mesh
Amplitude sweep	Limit LVR (%)	0.02 ± 0.01 ^a^	0.06 ± 0.00 ^b^	0.07 ± 0.00 ^c^	0.09 ± 0.00 ^d^
	Flow point (%)	20.34 ± 1.37 ^c^	2.17 ± 0.3 ^a^	3.76 ± 0.07 ^ab^	4.70 ± 0.02 ^b^
Frequency sweep	tanδ (at 10 rad/s)	0.45 ± 0.06 ^b^	0.21 ± 0.00 ^a^	0.19 ± 0.00 ^a^	0.18 ± 0.00 ^a^
	η* (at 10 rad/s; Pa·s)	3.94 ± 0.93 ^a^	5862 ± 273 ^b^	5539.30 ± 243 ^b^	10,923.33 ± 263 ^c^
Creep and recovery	$\gamma_{e}$ (%)	-	0.06 ± 0.02	0.05 ± 0.02	0.05 ± 0.01
	$\gamma_{v}$ (%)	-	0.12 ± 0.04	0.09 ± 0.04	0.05 ± 0.02
	$\gamma_{max}$ (%)	-	0.19 ± 0.03 ^b^	0.14 ± 0.02 ^ab^	0.10 ± 0.02 ^a^
	$\eta_{0\;}$(mPa·s)	-	(6.91 ± 1.85) × 10^9^	(7.59 ± 1.25) × 10^9^	(1.71 ± 1.25) × 10^10^
	J(t) (1/Pa)	-	(6.47 ± 3.50) × 10^−5 a^	(1.38 ± 0.24) × 10^−4 b^	(9.99 ± 1.6) × 10^−5 ab^

^a–d^ means different letters in the horizontal direction for % okara flour and particle size. The means are statistically significantly different (*p* < 0.05); each dataset analyzed by Tukey pairwise comparisons test.

**Table 3:** Physical properties of okara waffles with different percentages of okara flour.

Okara Flour (%)	0%	10%	20%	30%	40%
Width (cm)	6.22 ± 0.25 ^a^	8.12 ± 0.43 ^b^	6.28 ± 0.48 ^a^	7.70 ± 0.18 ^b^	6.42 ± 0.33 ^a^
Thickness (cm)	0.20 ± 0.00 ^a^	0.20 ± 0.00 ^a^	0.20 ± 0.00 ^a^	0.30 ± 0.01 ^a^	0.40 ± 0.01 ^b^
Spread Ratio	33.00± 7.32 ^c^	38.11 ± 4.24 ^c^	25.92 ± 4.44 ^b^	31.08 ± 8.85 ^b^	18.23 ± 4.04 ^a^
Water Activity (a_w_)	0.365 + 0.014 ^bc^	0.389 ± 0.016 ^c^	0.338 ± 0.011 ^b^	0.291 ± 0.001 ^a^	0.340 ± 0.004 ^b^
Moisture (%)	2.01 + 0.125 ^a^	5.1929 ± 0.0349 ^e^	4.5449 ± 0.0076 ^c^	4.0754 ± 0.0251 ^b^	4.6989 ± 0.0175 ^d^
L*	55.63 ± 1.62 ^ab^	62.32 ± 0.76 ^b^	60.26 ± 1.52 ^b^	58.40 ± 2.44 ^b^	53.78 ± 0.86 ^a^
a*	9.20 ± 0.24	9.37 ± 0.20	8.89 ± 0.32	8.60 ± 0.65	9.37 ± 0.27
b*	16.88 ± 0.17 ^b^	13.82 ± 0.33 ^a^	17.38 ± 0.82 ^b^	16.29 ± 1.50 ^b^	17.28 ± 0.04 ^b^
Hardness (g/s)	142.98 ± 14.78 ^a^	174.97 ± 16.22 ^a^	369.65 ± 39.06 ^d^	264.50 ± 16.24 ^b^	325.26 ± 15.48 ^c^

^a–e^ means different letters in the horizontal direction for % okara flour. The means are statistically significantly different (*p* < 0.05); each dataset analyzed by Tukey pairwise comparisons rest.

**Table 4:** Nutritional composition of okara flour and waffles (30% okara flour substitution).

Analyses	Okara Flour	Waffle 30% Okara Flour
Total energy (kcal/100 g)	387	457.88
Fat (g/100 g)	2.88	19.12
Protein (g/100 g)	35.47	10.77
Carbohydrate (g/100 g)	54.8	60.68
Sugar (g/100 g)	-	11.13
Sodium (mg/100 g)	-	628.56
Ash (g/100 g)	3.03	2.96
Moisture (%)	3.82	6.47

**Table 5:** Cholesterol and glucose adsorption capacities of okara flour and waffles (0% and 30% okara flour substitution) at pH 2 and pH 7.

Sample	CAC (mg/g) pH 2	CAC (mg/g) pH 7	GAC (mM/g)
Okara flour	366.24 ± 3.03 ^a^	313.84 ± 23.34 ^a^	99.27 ± 0.27 ^a^
Waffle 0% okara flour	639.24 ± 7.90 ^b^	641.04 ± 5.28 ^c^	182.72 ± 0.74 ^b^
Waffle 30% okara flour	640.07 ± 2.83 ^b^	618.71 ± 6.60 ^b^	183.82 ± 0.54 ^b^

^a–c^ means different letters in the horizontal direction for % okara flour. The means are statistically significantly different (*p* < 0.05); each dataset analyzed by Tukey pairwise comparisons test.

**Table 6:** Storage stability of okara waffles (30% okara flour) over 60 days.

Storage Period (Days)	Criteria	0	30	60
Water activity (a_w_)	-	0.378 ± 0.004 ^b^	0.354 ± 0.004 ^a^	0.371 ± 0.003 ^b^
Moisture (%)	-	4.640 ± 0.029 ^b^	4.492 ± 0.016 ^a^	4.932 ± 0.009 ^c^
L*	-	60.36 ± 1.34 ^ab^	58.52 ± 0.07 ^a^	61.38 ± 1.44 ^b^
a*	-	9.76 ± 0.32 ^ab^	10.08 ± 0.04 ^b^	9.27 ± 0.11 ^a^
b*	-	15.46 ± 0.57	15.60 ± 0.06	15.38 ± 0.18
Hardness (g/sec)	-	363.80 ± 11.47 ^b^	218.88 ± 10.45 ^a^	211.42 ± 6.09 ^a^
Yeasts (CFU/g)	<100	<10	-	<10
Molds (CFU/g)	<100	<10	-	60
*E. coli* (MPN/g)	<3	<3	-	<3
*C. perfringens* (CFU/g)	<100	<10	-	<10
*B. cereus* (CFU/g)	<1000	<100	-	<10
*S. aureus* (CFU/g)	<10	<10	-	<10
*Salmonella* spp. (/25 g)	ND	ND	-	ND

^a–c^ means different letters in the horizontal direction for each parameter (day of storage). The means are statistically significantly different (*p* < 0.05); each dataset analyzed by Tukey pairwise comparisons test. ND = not detected.

**Table 7:** Sensory test results of okara waffles using the 9-point hedonic scale.

Okara Flour (%)	Appearance	Soybean Flavor	Taste	Texture	Overall Liking	Accepted (%)	Not Accepted (%)
30%	7.57 ± 1.04	6.95 ± 1.73	7.02 ± 1.67	7.37 ± 1.50 ^b^	7.43 ± 1.41 ^b^	87	13
40%	7.63 ± 1.61	6.43 ± 1.50	6.50 ± 1.63	6.53 ± 1.74 ^a^	6.50 ± 1.57 ^a^	80	20

^a,b^ means different letters in the vertical direction for % okara flour. The means are statistically significantly different (*p* < 0.05); each dataset analyzed by independent *t*-tests.

**Table 8:** Cochran’s Q test results for sensory attributes of waffle samples.

Attribute	*p*-Values	Okara 0%	Okara 30%	Benchmark 1	Benchmark 2	Benchmark 3
Crispy	<0.0001	0.980 ^c^	0.740 ^ab^	0.740 ^ab^	0.920 ^bc^	0.700 ^a^
Hard	<0.0001	0.080 ^a^	0.700 ^b^	0.120 ^a^	0.280 ^a^	0.260 ^a^
Sweet	<0.0001	0.240 ^ab^	0.120 ^ab^	0.760 ^c^	0.300 ^b^	0.060 ^a^
Salty	<0.0001	0.640 ^c^	0.260 ^b^	0.040 ^a^	0.040 ^a^	0.060 ^ab^
Nutty	<0.0001	0.080 ^a^	0.640 ^b^	0.060 ^a^	0.040 ^a^	0.100 ^a^
Coconut	<0.0001	0.760 ^b^	0.180 ^a^	0.560 ^b^	0.040 ^a^	0.040 ^a^
Fruity	<0.0001	0.040 ^a^	0 ^a^	0.100 ^a^	0.800 ^c^	0.400 ^b^
Oily	<0.0001	0.520 ^c^	0.220 ^ab^	0.380 ^bc^	0.060 ^a^	0.180 ^ab^
Dry/Powdery	0.003	0.040 ^a^	0.220 ^b^	0.080 ^a^	0.080 ^a^	0.140 ^ab^
Plant-based	0.143	0.280	0.280	0.380	0.400	0.400
High protein	<0.0001	0.100 ^a^	0.560 ^b^	0.140 ^a^	0.080 ^a^	0.100 ^a^
High fiber	<0.0001	0.060 ^a^	0.400 ^b^	0.160 ^a^	0.160 ^a^	0.220 ^ab^
Gluten-free	<0.0001	0.260 ^a^	0.480 ^b^	0.500 ^b^	0.220 ^a^	0.240 ^a^
Vegan	0.271	0.580	0.500	0.580	0.640	0.580
Long shelf life	0.050	0.440	0.380	0.300	0.320	0.320
No additives	0.571	0.280	0.220	0.220	0.280	0.280
No preservatives	0.207	0.280	0.240	0.240	0.200	0.280

The table presents the *p*-values for each attribute; ^a–c^ means different letters in the horizontal direction for Sensory Attributes. The means are statistically significantly different (*p* < 0.05); each dataset analyzed by Tukey pairwise comparisons test.

## Data Availability

The original contributions presented in the study are included in the article, further inquiries can be directed to the corresponding author.
